# Hepatic granulomas as a manifestation of ANCA-associated vasculitis:a systematic review

**DOI:** 10.3389/fimmu.2026.1879137

**Published:** 2026-07-23

**Authors:** Tayssir Ben Achour, Nesrine Ben Hadj Dahman, Maysam Jridi, Fatma Said, Imed Ben Ghorbel, Ines Naceur, Monia Smiti

**Affiliations:** Department of Internal Medicine, La Rabta University Hospital, Faculty of Medicine of Tunis, University of Tunis El Manar, Tunis, Tunisia

**Keywords:** ANCA-associated vasculitis, EGPA, granulomatosis with polyangiitis, granulomatous hepatitis, hepatic granuloma, liver involvement, scoping review

## Abstract

**Background:**

ANCA-associated vasculitides (AAV) — granulomatosis with polyangiitis (GPA), eosinophilic granulomatosis with polyangiitis (EGPA), and microscopic polyangiitis (MPA) — are rare small-vessel autoimmune diseases. Liver involvement in AAV is uncommon and generally manifests as biochemical hepatitis; true hepatic granulomatosis is exceedingly rare and diagnostically challenging.

**Methods:**

We conducted a systematic scoping review following PRISMA-ScR guidelines and the Arksey & O’Malley framework, searching PubMed, Google Scholar, ScienceDirect, and Scopus without date restriction (through March 2026). A total of 7, 033 records were initially retrieved; after deduplication, title/abstract screening, and full-text review, five articles meeting strict inclusion criteria were included for qualitative synthesis.

**Results:**

Five published cases of hepatic granulomas in AAV patients with no confirmed confounding etiology were identified. All were GPA or EGPA; no case of pure MPA was documented. Three patients were female and two were male, with a mean age of 57.6 years. Liver histology revealed non-necrotizing epithelioid granulomas (n = 2), granulomatous inflammation with necrosis (n = 1), incomplete septal cirrhosis with vasculopathic changes (n = 1), and incidental calcified granulomas (n = 1). Immunosuppressive therapy with corticosteroids and/or cyclophosphamide achieved clinical and biochemical improvement in all treated patients.

**Conclusions:**

Hepatic granulomatosis is a rare but genuine extra-respiratory manifestation of AAV, most frequently reported in GPA. It may antedate the canonical ENT-pulmonary-renal triad, presenting as incidental hepatomegaly or unexplained liver function test elevation. Systematic exclusion of competing etiologies (sarcoidosis, tuberculosis, primary biliary cholangitis, drug-induced hepatitis) is mandatory before attributing granulomas to AAV. Liver biopsy remains pivotal in confirming the diagnosis. Immunosuppression is the therapeutic cornerstone, with generally favourable outcomes.

## 1 Introduction

Anti-neutrophil cytoplasmic antibody (ANCA)-associated vasculitides (AAV) encompass a group of rare, autoimmune, small-vessel diseases characterised by granuloma formation and/or necrotising inflammation of small vessels (1). The 2012 Chapel Hill Consensus Conference established a nosological framework comprising three main entities: granulomatosis with polyangiitis (GPA, formerly Wegener’s granulomatosis), microscopic polyangiitis (MPA), and eosinophilic granulomatosis with polyangiitis (EGPA, formerly Churg-Strauss syndrome) (2). The European prevalence of GPA is estimated at 120–140 cases per million, while EGPA prevalence ranges from 2 to 38 cases per million (3, 4).

GPA is characterised by necrotising granulomatous inflammation of the upper and lower respiratory tract and pauci-immune glomerulonephritis, with anti-proteinase-3 (PR3-ANCA) antibodies detected in the majority of patients. EGPA is distinguished by marked peripheral eosinophilia, asthma, and anti-myeloperoxidase (MPO-ANCA) antibodies in approximately 40% of cases. MPA shares pauci-immune vasculitis without prominent granuloma formation and is predominantly MPO-ANCA positive (1, 5). The clinical spectrum of AAV is broad, ranging from indolent localised disease to rapidly progressive multi-organ failure.

Liver involvement in AAV, while not among the canonical manifestations, is not negligible. A systematic analysis by Willeke et al. (2015) demonstrated liver function test (LFT) abnormalities in 49.4% of treatment-naive AAV patients during active disease, with GPA patients showing significantly higher transaminase, gamma-GT, and alkaline phosphatase levels compared to MPA or EGPA (6). Elevated LFT correlated with the Birmingham Vasculitis Activity Score (BVAS) and normalised after immunosuppressive induction. Yet, this biochemical hepatic involvement is broadly distinct from true histological hepatic granulomatosis.

Hepatic granulomas occur in approximately 3.6–6% of all liver biopsy specimens (7, 8). The differential diagnosis is wide: primary biliary cholangitis (PBC) accounts for nearly half of all cases in Western series, followed by sarcoidosis (~8–37%), infectious etiologies (tuberculosis, Bartonella, viral hepatitis), drug-induced granulomas (seen in 2.5–29% of series depending on clinical setting), and idiopathic causes (7, 9, 10). Vasculitis-attributable hepatic granulomatosis, specifically within the AAV spectrum, is vanishingly rare.

Indeed, only isolated case reports have described hepatic granulomas as a presenting or concomitant feature of AAV, and in many of these, confounding causes — sarcoidosis overlap, prior tuberculosis exposure, or drug-induced hepatitis — have not been convincingly excluded. The diagnostic challenge is further compounded by the fact that hepatic granulomas may precede the development of canonical AAV manifestations, thereby delaying diagnosis by months to years (11, 12).

Given the paucity and heterogeneity of available data, we conducted a systematic scoping review to catalogue all published cases of hepatic granulomas formally attributed to AAV, following strict inclusion criteria that excluded cases with plausible competing etiologies. Our aim was to characterise the clinical, serological, histopathological, and therapeutic features of this unusual hepatic manifestation, and to determine whether hepatic granulomatosis can be considered a genuine — if rare — organ manifestation of ANCA-associated disease.

## 2 Methods

### 2.1 Study design and framework

This study was designed as a systematic scoping review, conducted according to the PRISMA extension for scoping reviews (PRISMA-ScR) and the five-stage Arksey & O’Malley framework, which encompasses: (i) identifying the research question; (ii) identifying relevant studies; (iii) selecting studies; (iv) charting the data; and (v) collating, summarising, and reporting the results (13, 14).

The central research questions were: (1) What are the existing published cases documenting hepatic granulomas in patients with confirmed ANCA-associated vasculitis? (2) Can hepatic granulomatosis be considered an organ manifestation of AAV in the absence of secondary causes?

### 2.2 Search strategy

The systematic literature search was conducted across four electronic databases — PubMed/MEDLINE, Google Scholar, ScienceDirect, and Scopus — without starting-date restriction, with a final search date of 27 March 2026. For each database, both free-text and controlled vocabulary search terms were used (**Table 1**):

**Table 1:** Search strategy: free-text and controlled vocabulary terms used for each database.

Database	Free-text terms	Automated/advanced search string
PubMed	liver, hepatic, granuloma, ANCA vasculitis	(“liver granuloma*” OR “hepatic granuloma*”) AND (“ANCA” OR “anti-neutrophil cytoplasmic antibod*” OR “Antineutrophil cytoplasmic antibod*”)
Google Scholar	liver granulomas ANCA vasculitis	“liver granulomas” OR “hepatic granulomas” AND ANCA vasculitis
ScienceDirect	liver granuloma, ANCA vasculitis	(“liver granuloma*” OR “hepatic granuloma*”) AND (“ANCA” OR “anti-neutrophil cytoplasmic antibod*”)
Scopus	liver granuloma, ANCA	(“Liver granuloma*” OR “hepatic granuloma*”) AND ANCA

### 2.3 Inclusion and exclusion criteria

Studies of any design published in peer-reviewed journals, in any language, were eligible for inclusion. We included reports in which a patient met validated classification criteria for an AAV entity (GPA, MPA, or EGPA — per 2012 Chapel Hill, 2022 ACR/EULAR, or equivalent criteria) and had a histologically confirmed hepatic granuloma.

We excluded: (i) grey literature (conference abstracts without peer-reviewed publication, except when representing unique, otherwise unreported cases); (ii) cases with confirmed drug-induced granulomatous hepatitis; (iii) cases where sarcoidosis or mycobacterial infection was established as the primary diagnosis; (iv) cases in which the diagnosis of AAV was not clinically confirmed with laboratory and/or histological support; and (v) cases in which liver histology did not include explicit mention of granulomas.

### 2.4 Study selection and data extraction

After removing duplicates, two reviewers independently screened titles and abstracts. Full texts of potentially eligible articles were retrieved and assessed against the inclusion/exclusion criteria. Disagreements were resolved by consensus discussion. Quality assessment of case reports was performed using the CARE (CAse REport) guidelines checklist.

Data were extracted into a standardised form capturing: first author, year, country, journal, study type, patient demographics (age, sex), AAV diagnosis and classification criteria, ANCA serotype, hepatic presentation (symptoms, LFT profile, imaging), biopsy characteristics (granuloma morphology, necrosis, distribution), ancillary investigations to exclude confounders, treatment, and outcome.

**Figure 1:**
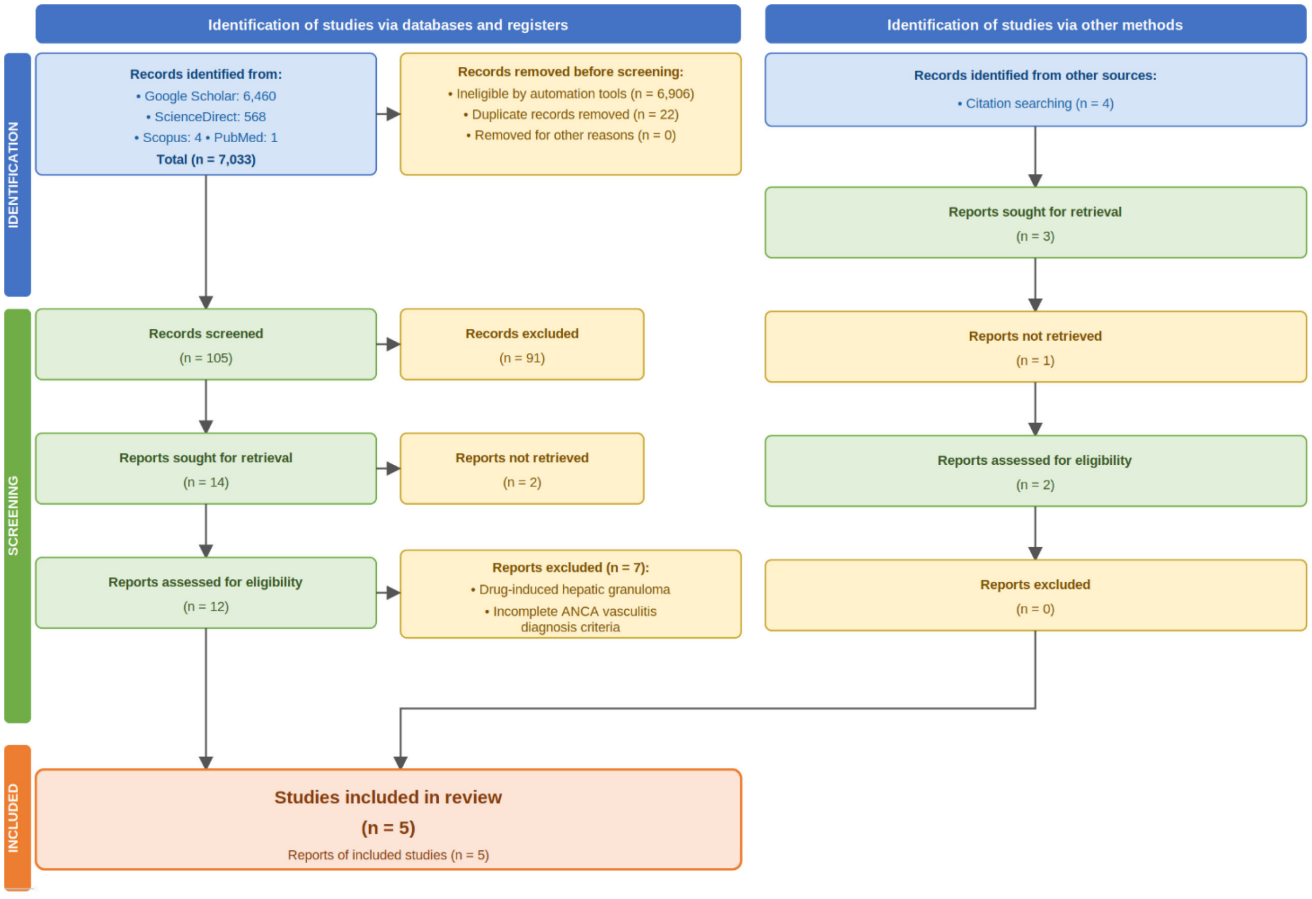
PRISMA 2020 flow diagram for new systematic reviews including searches of databases, registers, and other sources. Source: Page MJ, et al. BMJ 2021;372:n71.

The total number of records retrieved from all databases at initial search was 7, 033. After automated deduplication and advanced filtering, followed by manual title-abstract screening, 14 articles were selected for full-text review. Following the application of inclusion/exclusion criteria, five articles were retained for final qualitative synthesis. The study selection process is illustrated in the PRISMA-ScR flow diagram (**Figure 1**).

## 3 Results

### 3.1 Study characteristics

Five peer-reviewed publications reporting cases of hepatic granulomas in the context of ANCA-associated vasculitis satisfied the inclusion criteria (15–19). The articles spanned a period of 27 years (1999–2026) and originated from five different countries (USA, Tunisia, Greece, Japan, and the USA again in a conference-published case). Three cases involved GPA, one involved EGPA overlapping with Sjögren’s syndrome, and one described GPA-associated incomplete septal cirrhosis with vasculopathic hepatic injury. Across the five cases, three patients were female and two were male. Mean age at hepatic presentation was 57.6 years (range 47–75 years). A summary of study characteristics is provided in **Table 2**.

**Table 2:** Summary of included studies.

Author, year	Country	Disease	Age/sex	ANCA type	Hepatic finding	Histology (liver)
Schwartz/Niles 1999 (15)	USA	GPA	75 y/F	PR3-ANCA (cANCA)	Incidental calcified hepatic lesions on imaging	Calcified granulomas — histological confirmation limited (imaging only; likely prior TB exposure)
Zen 2002 (16)	Japan	GPA + Sjögren’s	47–73 y/F	pANCA	Incomplete septal cirrhosis, portal hypertension, encephalopathy	Autopsy: ISC with phlebosclerosis, narrowed portal veins — vasculopathic; no active granulomas at autopsy
Goritsas 2010 (17)	Greece	GPA	58 y/M	PR3-ANCA	Acute anicteric hepatitis (AST 781, ALT 512 U/L)	Mild non-specific lobular hepatitis; PAS+ Lafora-like inclusions; no definitive granulomas in liver
Ben Achour 2026 (18)	Tunisia	EGPA + Sjögren’s overlap	47 y/F	MPO-ANCA	Non-necrotising granulomas — presenting feature (incidental hepatomegaly at surgery)	Non-necrotising epithelioid and central giant-cell granulomas
Zaidi 2024 (19)	USA	GPA	51 y/F	PR3-ANCA (cANCA)	2.9 cm hepatic lesion (multifocal: liver + pancreas + kidney + lung)	EUS biopsy: necrotic material with acute inflammatory exudate and granulomatous inflammation

GPA, granulomatosis with polyangiitis; EGPA, eosinophilic GPA; ISC, incomplete septal cirrhosis; PR3, proteinase-3; MPO, myeloperoxidase; EUS, endoscopic ultrasound.

### 3.2 Detailed case descriptions

Case 1 — Schwartz & Niles (1999, NEJM) (15): A 75-year-old woman with a complex neurological presentation (pachymeningitis with granulomatous inflammation confirmed on dural biopsy, hydrocephalus, and progressive cognitive decline) was found to have incidental punctate hepatic calcifications on imaging. These findings were consistent with remote granulomatous disease, most likely prior subclinical tuberculosis exposure, in the context of established GPA. Although this case is often cited in the AAV-liver literature, the hepatic involvement was radiological only, without active biopsy-confirmed hepatic granulomas attributable to GPA. It is included here for completeness but should be interpreted as marginal evidence.

Case 2 — Zen et al. (2002, Liver) (16): A 47-year-old Japanese woman, diagnosed with GPA at age 57 (pANCA positive, lung biopsy showing granulomatous arteritis), developed progressive hepatic dysfunction at age 63. Laparoscopy revealed an uneven liver surface and umbilical vein dilatation. The patient developed portal hypertension, encephalopathy, and massive ascites and died of sepsis at age 73. Autopsy demonstrated incomplete septal cirrhosis (ISC) with characteristic phlebosclerosis and narrowing of portal veins — a vasculopathic pattern consistent with GPA affecting hepatic microvasculature. This is the first published case of ISC in the context of GPA, providing compelling — if indirect — evidence that small-vessel vasculitis can drive progressive hepatic fibrosis.

Case 3 — Goritsas et al. (2010, Journal of Medical Case Reports) (17): A 58-year-old Greek man presented with dry cough, fever, bilateral pulmonary alveolar infiltrates, and severe acute anicteric hepatitis (AST 781 U/L, ALT 512 U/L), in the absence of viral, toxic, or autoimmune hepatic aetiology. Lung biopsy confirmed necrotising granulomatous inflammation with Langhans giant cells; PR3-ANCA was strongly positive. Liver biopsy showed mild non-specific lobular hepatitis with PAS-positive Lafora-like inclusions in a large number of hepatocytes, but without definitive granulomas. Treatment with prednisone and cyclophosphamide induced remission of both pulmonary and hepatic disease, suggesting a vasculitis-driven mechanism of hepatocellular injury (likely hepatic vasculitis) rather than classic granuloma formation.

Case 4 — Ben Achour et al. (2026, Hepatology Forum) (18): A 47-year-old Tunisian woman underwent hiatal hernia surgery during which nodular hepatomegaly was incidentally discovered. Liver biopsy revealed non-necrotising epithelioid and central giant-cell granulomas. CT scan demonstrated perilymphatic pulmonary micronodules and bilateral hilar lymphadenopathies, initially raising the possibility of sarcoidosis. However, minor salivary gland biopsy confirmed Sjögren’s syndrome (Chisholm grade 3 sialadenitis), and immunological workup revealed positive MPO-ANCA with negative antinuclear antibodies. One year later, the patient developed asthma flare-ups and ENT symptoms; a nasal biopsy showed eosinophilic leukocytoclastic vasculitis, fulfilling 2022 ACR/EULAR criteria for EGPA. This case uniquely illustrates that: (i) hepatic granulomas may precede the diagnostic hallmarks of EGPA by over a year; (ii) AAV can overlap with other autoimmune diseases; and (iii) a systematic workup is mandatory before attributing hepatic granulomas to sarcoidosis.

Case 5 — Zaidi et al. (2024, CHEST Conference Abstract) (19): A 51-year-old American woman presented with abdominal pain and nausea. CT and MRI imaging revealed multifocal lesions in the pancreatic tail (3.6 cm), left hepatic lobe (2.9 cm), left renal pole (2.3 cm), and right upper pulmonary lobe (2.6 cm), raising primary concern for malignancy. Endoscopic ultrasound-guided biopsies of hepatic and pancreatic lesions demonstrated necrotic material with acute inflammatory exudate and granulomatous inflammation; robotic bronchoscopic cryo-biopsy of the pulmonary mass showed benign tissue with necrosis and loosely-formed granulomatous inflammation, highly suggestive of GPA. Past history included sinus surgery for chronic sinusitis with biopsy-proven granulomatous inflammation. This case highlights the deceptive malignancy-like presentation of GPA with hepatic granulomas and the critical role of tissue diagnosis across multiple organ sites.

### 3.3 Histopathological spectrum

The hepatic histological findings across the five cases spanned a spectrum reflecting distinct pathomechanisms (**Table 2**). True non-necrotising epithelioid granulomas — the classical paradigm of granulomatous hepatitis — were documented in only one case (Ben Achour 2026). Granulomatous inflammation with necrosis (Zaidi 2024) mirrors the necrotising pattern typical of GPA at extra-hepatic sites. In the Goritsas case, the absence of definitive granulomas in the liver biopsy, despite florid granulomatous pneumonitis on lung biopsy, points to hepatic vasculitis as the probable mechanism, rather than direct granuloma formation. The Zen case represents a distinct category altogether — vasculopathic fibrosis secondary to chronic GPA involvement of small hepatic vessels, culminating in incomplete septal cirrhosis without active granulomas.

### 3.4 Clinical and serological patterns

All cases of frank hepatic granulomas were associated with GPA (PR3-ANCA) or EGPA (MPO-ANCA); no case of MPA-associated hepatic granuloma was identified, consistent with the non-granulomatous histopathological profile of MPA. In the EGPA case, hepatic granulomas were the presenting manifestation, antedating canonical AAV features by approximately one year. In the GPA cases, hepatic involvement co-occurred with or followed pulmonary involvement. Multiorgan granulomatous involvement (lung, liver, pancreas, kidney) was documented in one GPA patient, illustrating the potentially systemic distribution of the granulomatous process.

Liver function test abnormalities, when documented, ranged from mild transaminase elevation to severe acute hepatitis (AST 781, ALT 512 U/L in the Goritsas case). Imaging findings included incidental calcifications, hepatomegaly with nodularity, and discrete space-occupying lesions mimicking malignancy or metastatic disease.

### 3.5 Differential diagnosis workup

A rigorous workup to exclude competing aetiologies is paramount given the broad differential diagnosis of hepatic granulomas. Across the included cases, sarcoidosis was systematically considered and excluded by clinical and radiological grounds, with the exception of the Ben Achour case where hilar lymphadenopathy initially suggested sarcoidosis before MPO-ANCA positivity and nasal biopsy findings confirmed EGPA. Tuberculosis was considered in the Schwartz/Niles case (incidental hepatic calcifications) but was not the active diagnosis. Drug-induced hepatic granulomas — common with antihypertensives, antirheumatic agents, antibiotics, and anticonvulsants — were excluded in all cases based on medication history and biopsy morphology (eosinophilic infiltrates, which favour drug aetiology, were not a dominant finding in liver biopsies in any case) (10).

### 3.6 Treatment and outcomes

In all cases where treatment was initiated, corticosteroids — alone or combined with cyclophosphamide — were the primary therapeutic strategy, consistent with current AAV induction guidelines (20). In the Goritsas case, prednisone and cyclophosphamide achieved remission of both pulmonary and hepatic disease, with normalisation of transaminases. In the Ben Achour case, oral corticosteroid therapy resulted in clinical improvement. The Zen case had an ultimately fatal outcome attributable to progressive portal hypertension and sepsis, illustrating the potential for severe, irreversible hepatic complications when AAV-related vascular injury goes unrecognised or inadequately treated.

## 4 Discussion

This scoping review represents, to our knowledge, the first systematic attempt to catalogue published cases of histologically confirmed hepatic granulomas attributed to AAV, applying explicit exclusion criteria for competing aetiologies. Our findings underscore several clinically important observations.

First, the extreme rarity of this manifestation: despite a search yielding over 7, 000 initial records, only five cases satisfied inclusion criteria over a period spanning nearly three decades. This rarity stands in contrast to the relatively common biochemical hepatic involvement in AAV — observed in approximately half of patients during active disease (6) — and underscores that transaminase elevation in AAV patients does not reliably predict histological granulomatosis.

Second, the histological heterogeneity is striking. True hepatic granulomas in AAV may be non-necrotising (EGPA) or necrotising (GPA), consistent with the known differential granuloma morphology between subtypes at other organ sites (5). Additionally, vasculopathic injury — as in the Zen ISC case — represents a distinct, potentially underdiagnosed mechanism by which AAV may cause chronic progressive hepatic disease without classic granuloma formation. This vasculopathic pathway, driven by small-vessel inflammation in hepatic portal tracts, may be more common than recognised, given that routine liver biopsy is not standard practice in AAV surveillance.

Third, hepatic granulomas may be the sentinel manifestation of EGPA, preceding canonical disease features by a year or more (Ben Achour 2026). This has significant diagnostic implications: clinicians performing liver biopsies for unexplained hepatomegaly or granulomatous hepatitis must routinely include ANCA serology in their investigation panel, particularly when sarcoidosis is not confirmed and respiratory symptoms are present or anticipated.

Fourth, the GPA-associated multifocal granulomatous presentation (Zaidi 2024), mimicking metastatic malignancy across liver, pancreas, kidney, and lung, illustrates the diagnostic pitfall of anchoring on the most feared diagnosis. In any patient with multifocal visceral lesions and no tissue diagnosis of malignancy, GPA should be a prominent differential, especially in the presence of ENT symptoms or prior sinus disease.

The pathophysiological mechanisms underlying hepatic granuloma formation in AAV are incompletely understood. In GPA, granulomas arise through a complex interplay of PR3-ANCA-driven neutrophil activation, T-cell-mediated granulomatous inflammation, and dysregulated macrophage differentiation into multinucleated giant cells (1). The same molecular machinery is presumably operative in hepatic tissue, though why granuloma formation occurs selectively in the liver in some GPA patients — while absent in others with comparable systemic inflammation — remains unexplained. In EGPA, eosinophilic tissue infiltration adds a further dimension; hepatic eosinophilic granulomas or epithelioid granulomas associated with eosinophilic vasculitis represent a distinct immunological subset.

Concerning treatment, the response to corticosteroids and immunosuppression in the documented cases aligns with broader AAV management principles. The 2021 ACR/EULAR guidelines for AAV induction recommend high-dose glucocorticoids combined with either rituximab or cyclophosphamide for severe/organ-threatening disease (20). Hepatic granulomatosis, given its potential for progressive fibrosis (as demonstrated in the Zen case), should arguably be classified as organ-threatening and managed accordingly.

This scoping review has several limitations. The evidence base is entirely constituted by case reports and one conference abstract, precluding any quantitative synthesis or generalisation of incidence estimates. Methodological heterogeneity in biopsy timing, reporting completeness, and exclusion of confounders varied across studies. Publication bias likely inflates the apparent novelty of these cases — unreported or misclassified cases of AAV-associated hepatic granulomas almost certainly exist. The retrospective nature of data collection prevents causal inference.

## 5 Conclusion

Hepatic granulomatosis is a rare but genuine manifestation of ANCA-associated vasculitis, documented principally in GPA and EGPA. It may precede the canonical respiratory-renal phenotype, particularly in EGPA, and may present as incidental hepatomegaly, unexplained hepatitis, or multifocal visceral lesions mimicking malignancy. Histological patterns range from classical non-necrotising epithelioid granulomas to necrotising granulomatous inflammation and vasculopathic fibrosis.

A systematic exclusion of competing aetiologies — sarcoidosis, mycobacterial infection, primary biliary cholangitis, and drug-induced granulomatous hepatitis — is mandatory before attributing hepatic granulomas to AAV. ANCA serology (both PR3 and MPO) should be part of the workup for any unexplained granulomatous hepatitis without a clear alternative diagnosis. Liver biopsy remains the cornerstone of diagnosis, and corticosteroid-based immunosuppression — combined with cyclophosphamide or rituximab in severe cases — is the therapeutic cornerstone.

Prospective registries and structured reporting of atypical AAV organ manifestations are needed to better characterise the prevalence, natural history, and optimal management of hepatic granulomatosis in this population.

## Data Availability

The original contributions presented in the study are included in the article/supplementary material. Further inquiries can be directed to the corresponding author.

## References

[1] Müller A Krause B Kerstein-Stähle A Comdühr S Klapa S Ullrich S . Granulomatous inflammation in ANCA-associated vasculitis. Int J Mol Sci. (2021) 22(12):6474. doi: 10.3390/ijms22126474 34204207 PMC8234846

[2] Jennette JC Falk RJ Bacon PA Basu N Cid MC Ferrario F . 2012 revised international chapel hill consensus conference nomenclature of vasculitides. Arthritis Rheum. (2013) 65:1–11. doi: 10.1002/art.37715 23045170

[3] Potentas-Policewicz M Fijolek J . Granulomatosis with polyangiitis: clinical characteristics and updates in diagnosis. Front Med. (2024). doi: 10.3389/fmed.2024.1369233 PMC11385631 39257888

[4] Yates M Watts RA Bajema IM Cid MC Crestani B Hauser T . EULAR/ERA-EDTA recommendations for the management of ANCA-associated vasculitis. Ann Rheum Dis. (2016) 75:1583–94. doi: 10.1136/annrheumdis-2016-209133 27338776

[5] Watts RA Robson J . Introduction, epidemiology and classification of vasculitis. Best Pract Res Clin Rheumatol. (2018) 32:3–20. doi: 10.1016/j.berh.2018.10.003 30526896

[6] Willeke P Schlüter B Limani A Becker H Schotte H . Liver involvement in ANCA-associated vasculitis. Clin Rheumatol. (2015) 35:387–94. doi: 10.1007/s10067-015-2882-5 25633652

[7] Drebber U Kasper HU Ratering J . Hepatic granulomas: histological and molecular pathological approach to differential diagnosis — a study of 442 cases. Liver Int. (2008) 28:828–34. doi: 10.1111/j.1478-3231.2008.01764.x 18312287

[8] Dourakis SP Saramandas V Alexopoulou A Kafiri G Deutsch M Koskinas J . Hepatic granulomas: a 6-year experience in a single centre in Greece. Eur J Gastroenterol Hepatol. (2007) 19:101–4. doi: 10.1097/01.meg.0000243882.09820.d2 17272993

[9] Ishak KG Zimmerman HJ . Drug-induced and toxic granulomatous hepatitis. Baillieres Clin Gastroenterol. (1988) 2:463–80. doi: 10.1016/0950-3528(88)90012-7 3044471

[10] Kleiner DE . Granulomatous liver diseases. In: Pathology of the Liver, 6th ed. Churchill Livingstone, London (2012).

[11] Poudel A Adhikari P Sundaresan V Jackson E Iskander B . Granulomatosis with polyangiitis presenting with hepatic involvement. Cureus. (2025) 17:e100357. doi: 10.7759/cureus.100357 41613716 PMC12851556

[12] Masiak A Drobińska A Zdrojewski Z . Hepatic involvement in GPA — diagnostic difficulties. PMC2025:5825971. 10.5114/reum.2017.72630 PMC5825971 29491541

[13] Arksey H O'Malley L . Scoping studies: towards a methodological framework. Int J Soc Res Methodol. (2005) 8:19–32. doi: 10.1080/1364557032000119616

[14] Tricco AC Lillie E Zarin W O'Brien KK Colquhoun H Levac D . PRISMA Extension for Scoping Reviews (PRISMA-ScR): checklist and explanation. Ann Intern Med. (2018) 169:467–73. doi: 10.7326/M18-0850 30178033

[15] Scully RE Mark EJ McNeely WF Ebeling SH . Case 9-1999: Wegener's granulomatosis with pachymeningeal granulomatous inflammation. N Engl J Med. (1999) 340:945–53. doi: 10.1056/NEJM199903253401208 10089189

[16] Zen Y Sunagozaka H Tsuneyama K . Incomplete septal cirrhosis associated with Wegener's granulomatosis. Liver. (2002) 22:388–93. doi: 10.1034/j.1600-0676.2002.01684.x 12390474

[17] Goritsas C Paissios NP Trigidou R Delladetsima J . Hepatic involvement in Wegener's granulomatosis: a case report. J Med Case Rep. (2010) 4:9. doi: 10.1186/1752-1947-4-9 20157433 PMC2821396

[18] Ben Achour T Saïd F Chérif A Jridi M Ghorbel IB Naceur I . Hepatic granulomas heralding eosinophilic granulomatosis with polyangiitis overlapping with Sjögren's syndrome. Hepatol Forum. (2026) 7:77–80. doi: 10.14744/hf.2025.45561 41589212 PMC12831983

[19] Zaidi W DeAnda S Murray C Saugstad A Moin A Razia D . Liver and pancreas involvement in granulomatosis with polyangiitis: a diagnostic challenge [conference abstract. CHEST. (2024) 166:A5773. doi: 10.12659/AJCR.945741 38826717

[20] Chung SA Langford CA Maz M Abril A Gorelik M Guyatt G . 2021 American College of Rheumatology/Vasculitis Foundation guideline for the management of antineutrophil cytoplasmic antibody-associated vasculitis. Arthritis Rheumatol. (2021) 73:1366–83. doi: 10.1002/art.41773 34235894 PMC12327957

